# The association of food consumption and macronutrient intake with dietary climate impact in Finland: considerations on the role of energy intake

**DOI:** 10.1017/S1368980025101730

**Published:** 2026-01-02

**Authors:** Laura Paalanen, Heli Tapanainen, Laura Sares-Jäske, Niina E. Kaartinen, Merja Saarinen, Liisa Valsta

**Affiliations:** 1 Finnish Institute for Health and Welfare (THL), P.O. Box 30, FI-00271 Helsinki, Finland; 2 Natural Resources Institute Finland (Luke), P.O. Box 2, FI-00791 Helsinki, Finland

**Keywords:** Sustainability, Food consumption, Energy intake, Dietary climate impact, Sociodemographic differences

## Abstract

**Objective:**

To study (1) the differences in dietary climate impact between sociodemographic groups, (2) the differences in food consumption and macronutrient intake as absolute amounts and in relation to energy intake by dietary climate impact level and (3) food groups as contributors of dietary climate impact.

**Design:**

Food consumption and energy and macronutrient intakes were calculated based on two non-consecutive 24-hour dietary recalls. Dietary climate impact was calculated using national coefficients produced with life cycle assessment. Regression analysis was used to test the mean differences between sociodemographic groups and sex-specific dietary climate impact tertiles.

**Setting:**

Finnish national food consumption survey FinDiet 2017.

**Subjects:**

In total, 565 men and 682 women (age 18–74) after exclusion of energy under-reporters.

**Results:**

The mean daily dietary climate impact was higher in men than in women (5·6 *v.* 4·0 kg CO_2_eq) and in younger age group (18–44 years) than in older age group (65–74 years). The association of food consumption and dietary climate impact was mainly different for food consumption as absolute amounts (g/d) and in relation to energy (g/MJ). In relation to energy, the consumption of animal-based foods was higher and plant-based foods lower in the highest dietary climate impact tertile compared with the lowest tertile. Red and processed meat was a major contributor to dietary climate impact.

**Conclusion:**

Our study emphasises the importance of considering food consumption and nutrient intake both as absolute amounts and in relation to energy intake. Our findings support the advantages of plant-based diets in being both healthier and more climate-friendly.

The benefits of plant-based diets for the health of both humans and the planet have been widely confirmed^(1–4)^. Diets must change to promote health and well-being of humans and to avoid exceeding planetary boundaries. This is increasingly acknowledged in recent food-based dietary guidelines^(5–8)^. The updated Nordic Nutrition Recommendations 2023, including food-based dietary guidelines, are primarily based on health, but environmental aspects are integrated into the recommendations more extensively than previously^(6)^. The Nordic Nutrition Recommendations recommend predominantly plant-based diets with limited intake of red meat and poultry and minimal intake of processed meat. The recent update of the Finnish National Nutrition Recommendations, published in 2024, is based on the Nordic recommendations and similarly addresses the environmental aspects in the food-based dietary guidelines but additionally takes the local food culture of Finland into account^(9)^.

Food systems are one of the major contributors to anthropogenic global warming with an estimated share of about one third of global greenhouse gas emissions (GHGEs)^(10,11)^. Nearly half of the GHGE from food systems is caused by crop and livestock production, about one fifth from land-use change and about one third from pre- and post-production processes, such as food manufacturing, retail, household consumption and food disposal^(12)^.

Although many studies based on actual food consumption data suggest an association between healthier diets and lower dietary GHGEs, i.e. climate impact^(13–17)^, not all studies confirm this association^(18–20)^. For example, in a study based on self-selected diets of French adults, high-nutritional-quality diets had significantly higher dietary climate impact after the GHGEs were adjusted for energy intakes^(19)^. On the other hand, a study from the Netherlands found that the consumption of both animal-based and plant-based foods with no adjustment for energy intake was higher in the high dietary climate impact group compared with the low climate impact group^(21)^. Under-reporting was, however, found to be more common in the low climate impact group, which suggests that the actual dietary GHGEs of the low climate impact group may have been higher than estimated.

Overall, in addition to the type of food consumed, both the amounts of food consumed and the energy intake are correlated with the dietary climate impact. Therefore, when the dietary climate impact is high, it is more likely that dietary recommendations are achieved for foods and nutrients for which exceeding a certain amount of food or nutrient is recommended. This may apply to, for example, achieving the recommended levels of fruit and vegetable consumption or fibre intake. Therefore, considering food consumption or nutrient intake adjusted for total energy intake might increase understanding of the relationship between the nutritional quality and environmental impacts of diets while considering the absolute amounts refers to actualised nutrient intakes and dietary environmental impacts.

In addition to accounting for energy intake, consideration of energy under-reporting is essential. A study from the UK examined the association between diet quality and dietary climate impact assessed with three different diet scores^(22)^. Energy misreporting was evaluated based on the ratio of reported energy intake to estimated energy requirement. The analyses revealed inconsistent associations for the different diet quality scores when energy misreporting was not adjusted for. After adjusting for energy misreporting, an inverse association between diet quality and dietary climate impact was seen for all three diet quality scores. The analyses were also performed excluding the energy misreporters. Among plausible reporters, the association between diet quality and dietary climate impact was inverse, irrespective of adjustments. Thus, energy under-reporting/misreporting may complicate the analyses and interpretations and requires attention in studies on dietary climate impact.

Different methods to account for energy intake, as well as including/excluding misreporters, were compared in a recent study from Sweden examining the relation between the dietary climate impact and micronutrient intake^(23)^. The different methodologies included in the comparison lead to varying results and conclusions. Currently, the means of taking these issues into account are not established.

These methodological concerns also apply to the study questions on whether the dietary climate impact varies between sociodemographic groups, which has been examined in some studies^(21,24,25)^. The studies show higher dietary climate impact among men than among women^(13–16,21,24–26)^, which is not surprising given the higher energy requirement of men compared to women. The evidence is less clear for other sociodemographic groups^(14,24–28)^.

Red and processed meat and other animal-based products have been estimated to be among the major contributors to dietary climate impact^(21,22,24,26,29)^. Furthermore, the contribution of different foods to the dietary climate impact has been analysed in the Netherlands for adults and children by sex, and meat and cheese contributed about 40 % of the dietary climate impact in all included subgroups, i.e. among girls, boys, women and men^(21)^.

Examining how dietary climate impacts are distributed across different sociodemographic groups helps to identify the population groups with the highest impacts. This information is useful when public health strategies and measures pursuing reductions in climate impacts are planned. Simultaneously, the measures should aim at improving the quality of diets in the whole population. In addition, knowledge of food groups with major contribution to the dietary climate impact nationally is essential to target the dietary change interventions effectively. Our aims were to study (1) the differences in dietary climate impact between different sociodemographic groups in Finland, (2) the differences in food consumption and macronutrient intake as absolute amounts and in relation to energy intake by dietary climate impact level and (3) the contribution of each food ingredient group (%) to the total dietary climate impact by sex, age and dietary climate impact level.

## Methods

### Study population

#### FinDiet 2017 Survey

Data from the national FinDiet 2017 Survey were used. The FinDiet 2017 was a sub-sample of the national FinHealth 2017 Study, a health examination survey carried out in Finland between January and May 2017^(30–32)^. For the FinHealth 2017 Study, a representative sample of adults aged 18 years and above (*n* 10 247) was drawn from the Population Register. A sub-sample of those aged 18–74 years (*n* 3099, 30 % of the FinHealth 2017 Study sample) was invited to participate in the FinDiet 2017 Survey. Of these, 59 % participated in the health examination and were eligible for the dietary data collection. Diet was assessed by two non-consecutive 24-hour dietary recalls carried out by trained dietary interviewers. After exclusion of persons with incomplete or missing dietary data (*n* 159 for whom two accepted recalls were not achieved), 1655 adults aged 18–74 years (53 % of the invited) formed the FinDiet 2017 Survey data (780 men and 875 women).

Foods consumed by the study participants were disaggregated into their ingredients using standard recipes of the national food composition database Fineli®^(33)^. The average daily consumption of food ingredients and intake of energy and macronutrients were calculated using an in-house dietary software^(34)^ and Fineli®. The energy intake was calculated as MJ/d and the intake of macronutrients as energy percentage (E%). The intake of fibre was calculated in relation to energy (g/MJ). Food ingredient consumption was calculated both as absolute amounts (g/d) and in relation to energy (g/MJ).

For this study, energy under-reporters were excluded (215 men and 192 women) (more information below in chapter *Dietary climate impact tertiles)*. The energy under-reporters were identified following the EU Menu methodology^(35)^. In addition, one person was excluded due to missing background information. After these exclusions, the data of the present study included 1247 respondents (565 men and 682 women).

#### Food ingredient groups

Fourteen food ingredient groups, classified according to the Fineli® food grouping system, were chosen to represent major animal-based and plant-based food groups: (1) vegetables and fruit (including berries), (2) potatoes, (3) legumes, (4) nuts and seeds, (5) beef, (6) pork, (7) red and processed meat (including beef, pork, lamb, game, offal, sausages and cold cuts), (8) poultry, (9) fish and seafood, (10) liquid dairy products (including milk and most sour milk products such as yogurt), (11) cheese (matured cheeses), (12) butter and butter-containing spreads, (13) vegetable oil and margarine (including salad dressings and mayonnaise) and (14) cereals. Beef and pork are included in the red and processed meat group but also reported as separate food groups because of their different climate impacts. Liquid dairy products, cheese and butter and butter-containing spreads were kept as separate indicators as they may be related to somewhat different dietary patterns.

The selected 14 food ingredient groups do not cover the whole diet but serve as indicators of major animal-based and plant-based food groups. However, all food ingredient groups and not only the fourteen selected indicator groups are included in the dietary climate impact calculation.

#### Dietary climate impact calculation

In this paper, the dietary GHGE sum is referred to as the dietary climate impact. The dietary climate impact was calculated from food consumption data (FinDiet 2017 Survey), covering the whole diet of the participants, using climate impact (GHGE) coefficients for foods (kg CO_2_ eq/kg food). Climate impact coefficients produced with the life cycle assessment were linked to food ingredient groups of the food composition database Fineli®. Coefficients for food ingredient groups were retrieved from the Natural Resources Institute Finland’s FoodMin dietary model^(36)^ and were based on the institute’s previous life cycle assessment studies and scientific literature. The FoodMin dietary model consists of life cycle assessment-based climate impact estimations for ninety-one food product groups and following the food categorisation used in the FinDiet 2017 survey and Fineli® food composition database. For each product group, minimum and maximum values were set based on the available data, and the average of these values was used to calculate estimates for this study. The FoodMin model contains coefficients for both Finnish and imported products. For this study, they were combined into one value for each food group based on self-sufficiency rates^(36)^, thus representing foods consumed in Finland. Further, only climate impacts derived from food production were accounted for in FoodMin model, excluding consumption-related activities such as cooking, shopping, food storage and consumer’s food waste, as well as part of manufacturing phases and packaging. However, these phases are roughly similar for all diets.

Individual-level dietary climate impact covering the whole diet, i.e. GHGE sum/d (kg CO_2_ eq/d), was estimated by multiplying the consumption of each food ingredient group (g/d, mean of the two dietary recalls) by the respective group’s climate impact coefficient, and by summing the climate impacts of all consumed ingredient groups. These absolute GHGE sums/d were used in all analyses, i.e. GHGE sums were not adjusted for total energy intake.

#### Dietary climate impact tertiles

Preliminary inspection of dietary climate impacts was performed with data including under-reporters. The preliminary analyses revealed that the proportion of under-reporters was notable among participants with low dietary climate impact; about half of the subjects in the lowest climate impact group were classified as energy under-reporters. Therefore, we examined the correlation between energy intake and dietary climate impact, and the Spearman’s rank correlation coefficient was 0·63 for women and 0·64 for men. Based on these preliminary analyses, we excluded under-reporters as described under chapter *FinDiet 2017 Survey* (28 % of men, 22 % of women) from the final analysis data set. After exclusions, the respondents were divided into sex-specific climate impact tertiles based on dietary climate impact per day.

#### Background information

Data on sex and age were obtained from the sampling frame, information for the sociodemographic variables (education and income) was obtained from the FinHealth 2017 Study questionnaires and information on residential area was obtained from the Population Register Centre (coordinates of the residence of the participants) and Statistics Finland (categorisation of urbanisation level of residential area based on these coordinates).

The three educational categories used here – ‘low’, ‘middle’ and ‘high’ education level – were created by dividing self-reported number of years of full-time studying (including primary school) into tertiles by sex and birth year. Questions on total household income before tax deductions during the previous year, and on number of adult and underage household members, were utilised to determine the income group. The household income question contained ten categories: from ‘less than EUR 15 000’ and ‘EUR 15 001–25 000’ to ‘more than EUR 90 000’ income per year. For this study, the upper limits of the bottom nine original response categories (e.g. EUR 15 000 for the lowest category and EUR 25 000 for the next category), and the lower limit of the highest category (i.e. EUR 90 000) multiplied by two, were divided by weighted sum of household members, assigning a weight of 1·0 to the first adult, 0·7 to additional adults and 0·5 to underage household members^(37)^. The resulting individual values were grouped into sex-specific quartiles, which in turn were combined into three groups: ‘lowest quartile’, ‘middle quartiles’ (2.–3. quartiles combined) and ‘highest quartile’. Urbanisation levels were categorised as follows: ‘urban’ (urban areas), ‘semi-urban’ (areas near urban areas and rural centres) and ‘rural’ (remote rural areas). The proportions of study population in the categories of these sociodemographic variables are presented in Table 1.

**Table 1:** The numbers and proportions of participants and means and CIs of dietary climate impact (greenhouse gas emission sum) in age, education, income and urbanisation level groups by sex (FinDiet 2017 survey, *n* 1247, under-reporters excluded)

	Men					Women				
			Dietary climate impact (kg CO_2_ eq/d)					Dietary climate impact (kg CO_2_ eq/d)		
	*n*	%	Mean (kg CO_2_ eq/d)	95 % CI	General test	*n*	%	Mean (kg CO_2_ eq/d)	95 % CI	General test
All	565		5·6	5·4, 5·8		682		4·0	3·9, 4·1	
Age (years)					< 0·001^*^					< 0·001^*^
18–44	210	37 %	6·2	5·8, 6·6		241	35 %	4·3	4·1, 4·5	
45–64	208	37 %	5·4	5·1, 5·7		247	36 %	3·9	3·8, 4·1	
65–74	147	26 %	4·3	4·0, 4·6		194	28 %	3·5	3·3, 3·6	
Education					NS					NS
Low	193	34 %	5·5	5·1, 5·8		197	29 %	3·9	3·7, 4·1	
Middle	178	32 %	5·9	5·4, 6·4		225	33 %	4·2	3·9, 4·4	
High	191	34 %	5·4	5·0, 5·8		250	37 %	4·0	3·8, 4·2	
Income					NS					NS
Lowest quartile	145	26 %	5·7	5·2, 6·2		131	20 %	4·0	3·7, 4·4	
Middle (2.–3. quartiles)	290	52 %	5·4	5·1, 5·7		322	49 %	4·0	3·8, 4·1	
Highest quartile	123	22 %	5·9	5·3, 6·5		206	31 %	4·1	3·9, 4·3	
Urbanisation level					NS					NS
Urban	312	55 %	5·7	5·3, 6·0		426	62 %	4·1	3·9, 4·2	
Semi-urban	154	27 %	5·7	5·3, 6·2		146	21 %	4·0	3·8, 4·3	
Rural	99	18 %	5·0	4·7, 5·3		110	16 %	3·9	3·7, 4·1	

* Pairwise comparison: All pairwise comparisons for differences between age groups were statistically significant (*P* < 0·05).

### Statistical methods

All analyses were performed separately for men and women. Survey weights were used to adjust for non-participation bias and to improve the generalisability of the results to the Finnish adult population^(38)^.

Mean consumption of food ingredients or intake of macronutrients was calculated using the mean of the two days for each subject. Regression analysis was used to test the mean differences between sociodemographic groups as well as between dietary climate impact tertiles. Age was included as a covariate in the regression models. Consumption or intake data were transformed prior to regression analysis using cubic root transformation to achieve normality. For pair-wise tests, multiple comparisons were taken into account using the Tukey-Kramer adjustment. For some episodically consumed food groups, it was not possible to transform the consumption data into a normal distribution. For these, the Kruskal-Wallis non-parametric test was used. Pair-wise comparisons were not performed for non-parametric tests. Consumption of food groups and macronutrients both as absolute amounts (g/d) and in relation to energy (g/MJ or E%) was used in the analyses.

**Figure 1:**
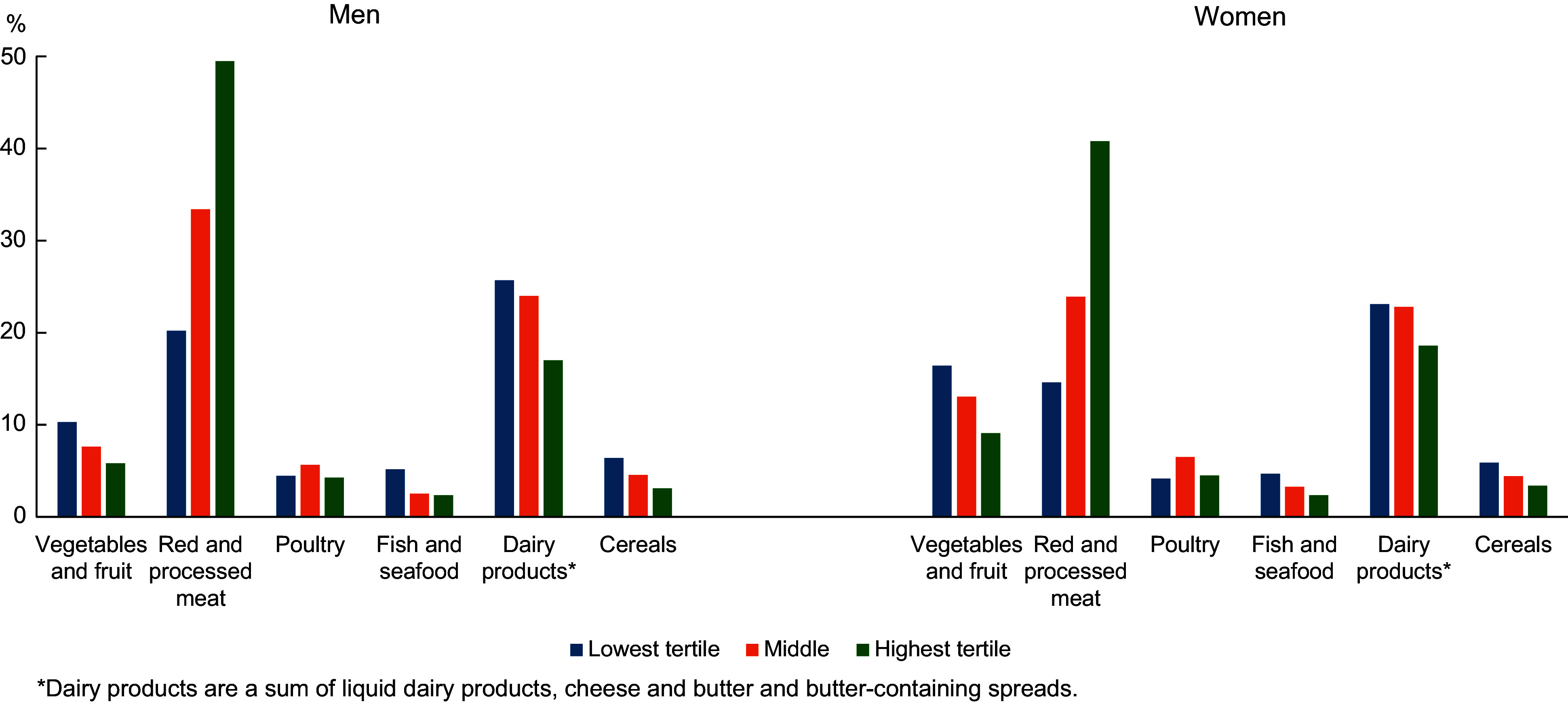
Proportion of food ingredient groups as contributors of dietary climate impact in dietary climate impact tertiles by sex (FinDiet 2017 Survey, *n* 1247, under-reporters excluded).

The contribution of food ingredient groups to the total dietary climate impact (per cent of the total) was calculated by sex as well as by dietary climate impact tertiles and age groups (separately for men and women). To cover all food ingredient groups, food ingredient groups which were not among the fourteen food ingredient groups selected for reporting were combined to group ‘other’. The components of red and processed meat as contributors of dietary climate impact were also examined separately: (1) beef, (2) pork and (3) other red meat or processed meat (including lamb, game, offal as well as sausages and cold cuts). For Figure 1 and see online supplementary material, Supplemental Figure 1, the sum of (1) liquid dairy products, (2) cheese (matured cheeses) and (3) butter and butter-containing spreads was calculated. This sum variable ‘dairy products’ does not, however, cover all dairy products and excludes, for example, cream, ice cream, cream cheese and quark.

## Results

### Differences in dietary climate impact between sociodemographic groups

The mean dietary climate impact was higher among men than among women, 5·6 *v*. 4·0 kg CO_2_ eq/d, respectively, and higher in younger age groups compared to older age groups (Table 1). In the youngest age group (18–44 years), the climate impacts were 6·2 and 4·3 kg CO_2_ eq/d, respectively, while in the oldest age group (65–74 years), the respective figures were 4·3 and 3·5 kg CO_2_ eq/d. No significant differences by education, income or urbanisation level were seen.

### Food consumption by dietary climate impact tertiles

The mean consumption of foods classified into fourteen food ingredient groups is presented both as absolute amounts per day (g/d) and in relation to energy (g/MJ) in sex-specific dietary climate impact tertiles (Tables 2 and 3).

**Table 2:** Means and CIs of consumption of food ingredient groups (g/d and g/MJ) in men by dietary climate impact (greenhouse gas emission sum) tertiles (FinDiet 2017 survey, *n* 565, under-reporters excluded)

	Men (g/d)									Men (g/MJ)							
		Climate impact tertile								Climate impact tertile							
	All men	Lowest (1)		Middle (2)		Highest (3)		General test	Pairwise comparison	Lowest (1)		Middle (2)		Highest (3)		General test	Pairwise comparison
	Mean	Mean	95 % CI	Mean	95 % CI	Mean	95 % CI	*P* value	Sign. diff.^*^	Mean	95 % CI	Mean	95 % CI	Mean	95 % CI	*P* value	Sign. diff.^*^
Vegetables and fruit^†^	332·6	325·6	294·8, 356·4	314·4	281·0, 347·8	353·3	313·8, 392·7	NS		37·7	34·3, 41·1	31·4	28·1, 34·8	29·8	26·4, 33·1	0·002	1 > 2, 1 > 3
Potatoes	91·4	82·3	70·9, 93·6	95·5	81·8, 109·1	94·8	81·4, 108·2	NS	^‡^	9·6	8·3, 10·9	9·4	8·1, 10·7	7·8	6·7, 8·9	NS	^‡^
Legumes	14·3	14·7	9·8, 19·6	10·3	5·6, 15·1	17·4	9·7, 25·1	NS	^‡^	1·7	1·1, 2·2	0·9	0·6, 1·3	1·4	0·9, 2·0	NS	^‡^
Nuts and seeds	7·6	7·6	4·1, 11·1	7·0	4·0, 9·9	8·0	5·5, 10·5	NS	^‡^	0·8	0·5, 1·2	0·7	0·4, 0·9	0·6	0·5, 0·8	NS	^‡^
Red and processed meat	148·7	69·4	57·2, 81·6	142·2	130·0, 154·5	214·6	198·3, 230·9	< 0·00001	1 < 2, 1 < 3, 2 < 3	8·2	6·9, 9·5	13·9	12·9, 15·0	18·1	16·6, 19·6	< 0·00001	1 < 2, 1 < 3, 2 < 3
Beef	34·4	3·2	1·6, 4·8	14·6	11·6, 17·7	74·9	64·3, 85·5	< 0·00001	^‡^	0·4	0·2, 0·6	1·6	1·3, 2·0	6·5	5·6, 7·4	< 0·00001	^‡^
Pork	39·2	30·7	20·9, 40·5	42·1	32·9, 51·4	43·3	33·4, 53·1	NS	^‡^	3·5	2·5, 4·5	4·0	3·1, 4·8	3·5	2·7, 4·3	NS	^‡^
Poultry	47·8	27·7	18·9, 36·6	50·8	39·8, 61·9	60·6	45·8, 75·4	0·003	^‡^	3·1	2·2, 4·1	5·2	4·0, 6·4	5·0	3·8, 6·2	0·03	^‡^
Fish and seafood	38·6	43·9	34·3, 53·6	29·9	23·1, 36·7	41·9	23·2, 60·7	0·03	^‡^	5·1	4·0, 6·1	2·9	2·3, 3·6	3·2	2·0, 4·4	0·001	^‡^
Liquid dairy products	412·3	330·8	274·0, 387·6	439·1	384·2, 494·0	451·6	390·3, 512·9	0·01	1 < 2	39·2	32·5, 45·9	43·0	37·7, 48·2	36·5	32·1, 40·8	NS	
Cheese^§^	37·3	25·4	21·6, 29·3	34·9	30·6, 39·3	48·4	41·9, 54·8	< 0·00001	^‡^	3·0	2·5, 3·4	3·4	3·0, 3·8	4·0	3·5, 4·5	0·005	^‡^
Butter and butter- containing spreads	20·7	17·8	14·5, 21·1	23·2	19·8, 26·6	20·8	16·7, 24·8	NS		2·0	1·7, 2·4	2·3	2·0, 2·6	1·8	1·4, 2·1	NS	
Vegetable oil and margarine	32·8	28·3	25·1, 31·4	30·8	27·4, 34·3	38·0	32·1, 43·8	0·01	1 < 3	3·3	2·9, 3·7	3·0	2·7, 3·2	3·1	2·7, 3·4	NS	
Cereals^||^	166·1	156·6	148·0, 165·2	163·9	152·0, 175·8	175·2	155·7, 194·8	NS		18·2	17·2, 19·2	15·9	15·0, 16·7	14·3	13·2, 15·3	< 0·00001	1 > 2, 1 > 3, 2 > 3

Sign. diff., significant difference.

* Considered significantly different with group rankings as indicated, if for the general test *P* < 0·05 and for pair-wise comparison *P* < 0·05.

† Including berries.

‡ Pair-wise comparisons not produced for food ingredient groups where the non-parametric general test had to be used.

§ Only matured cheeses included.

|| Including wheat, barley, oat, rye, rice, starch, other cereals and cereal bars.

**Table 3:** Means and CIs of consumption of food ingredient groups (g/d and g/MJ) in women by dietary climate impact (greenhouse gas emission sum) tertiles (FinDiet 2017 survey, *n* 682, under-reporters excluded)

	Women (g/d)									Women (g/MJ)							
		Climate impact tertile								Climate impact tertile							
	All women	Lowest (1)		Middle (2)		Highest (3)		General test	Pairwise comparison	Lowest (1)		Middle (2)		Highest (3)		General test	Pairwise comparison
	Mean	Mean	95 % CI	Mean	95 % CI	Mean	95 % CI	*P* value	Sign. diff.^*^	Mean	95 % CI	Mean	95 % CI	Mean	95 % CI	*P* value	Sign. diff.^*^
Vegetables and fruit^†^	403·2	410·8	366·9, 454·7	411·1	377·2, 445·0	389·4	358·4, 420·5	NS		57·7	52·8, 62·6	52·0	48·0, 55·9	43·4	40·1, 46·8	0·002	1 > 3, 2 > 3
Potatoes	62·7	59·6	50·3, 69·0	61·4	53·4, 69·4	66·5	58·2, 74·7	NS	^‡^	8·7	7·3, 10·1	7·7	6·7, 8·7	7·4	6·5, 8·3	NS	^‡^
Legumes	12·5	16·6	11·9, 21·4	10·0	7·1, 12·9	11·2	7·4, 15·0	NS	^‡^	2·3	1·7, 2·9	1·3	0·9, 1·6	1·2	0·8, 1·6	NS	^‡^
Nuts and seeds	10·6	12·3	9·6, 15·1	9·0	7·0, 11·0	10·6	8·2, 13·0	NS	^‡^	1·7	1·3, 2·0	1·1	0·8, 1·3	1·1	0·9, 1·4	NS	^‡^
Red and processed meat	76·5	40·1	34·7, 45·6	66·0	59·1, 73·0	117·5	109·2, 125·8	< 0·00001	^‡^	6·0	5·2, 6·8	8·6	7·7, 9·6	13·5	12·5, 14·5	< 0·00001	^‡^
Beef	19·5	2·5	1·5, 3·5	10·4	8·1, 12·6	42·6	36·7, 48·5	< 0·00001	^‡^	0·4	0·2, 0·6	1·5	1·1, 1·8	5·0	4·3, 5·7	< 0·00001	^‡^
Pork	19·1	18·6	14·0, 23·2	18·9	14·1, 23·6	19·7	14·7, 24·8	NS	^‡^	2·8	2·1, 3·5	2·3	1·8, 2·9	2·2	1·7, 2·7	NS	^‡^
Poultry	36·8	20·8	14·9, 26·7	43·8	34·6, 53·0	44·3	35·4, 53·2	0·001	^‡^	3·0	2·2, 3·9	5·6	4·4, 6·8	4·9	3·9, 5·9	0·007	^‡^
Fish and seafood	29·5	31·3	25·5, 37·0	29·2	22·9, 35·4	28·2	23·1, 33·2	NS	^‡^	4·5	3·7, 5·3	3·6	2·8, 4·3	3·1	2·5, 3·6	NS	^‡^
Liquid dairy products	323·5	253·2	222·3, 284·1	333·8	294·8, 372·8	375·0	336·9, 413·1	0·00001	1 < 2, 1 < 3	36·3	31·9, 40·7	42·3	37·5, 47·0	41·2	37·0, 45·4	0·01	1 < 2, 1 < 3
Cheese^§^	24·8	16·7	14·5, 18·9	24·4	21·9, 27·0	32·0	28·7, 35·4	< 0·00001	^‡^	2·5	2·1, 2·8	3·1	2·8, 3·5	3·5	3·2, 3·9	0·00001	^‡^
Butter and butter-containing spreads	14·8	12·6	10·5, 14·6	15·3	13·0, 17·5	16·3	14·1, 18·5	0·009	1 < 3	1·8	1·5, 2·1	1·9	1·6, 2·1	1·7	1·5, 2·0	NS	
Vegetable oil and margarine	22·5	20·7	18·4, 23·0	21·6	19·0, 24·2	24·8	22·4, 27·2	0·04	NS	2·9	2·6, 3·2	2·7	2·4, 3·1	2·7	2·4, 3·0	NS	
Cereals^||^	121·4	114·3	108·3, 120·4	116·6	110·2, 122·9	131·8	126·0, 137·6	0·0008	1 < 3, 2 < 3	16·4	15·5, 17·2	14·6	13·9, 15·4	14·5	13·9, 15·1	0·003	1 > 2, 1 > 3

Sign. diff., significant difference.

* Considered significantly different with group rankings as indicated, if for the general test *P* < 0·05 and for pair-wise comparison *P* < 0·05.

† Including berries.

‡ Pair-wise comparisons not produced for food ingredient groups where the non-parametric general test had to be used.

§ Only matured cheeses included.

|| Including wheat, barley, oat, rye, rice, starch, other cereals and cereal bars.

Higher climate impact was mostly associated with higher absolute consumption of food groups (g/d). This applied to red and processed meat, beef, poultry, liquid dairy products, cheese and vegetable oil and margarine among both sexes, and to butter and butter-containing spreads as well as cereals among women. For example, the consumption of red and processed meat in the lowest and highest climate impact tertiles was 69 g/d and 215 g/d in men, respectively, and 40 g/d and 118 g/d in women, respectively. As an exception, fish and seafood consumption among men was lowest in the middle dietary climate impact tertile.

The relationship of food consumption in relation to energy (g/MJ) and dietary climate impact varied between animal-based and plant-based foods. The consumption of red and processed meat, beef, poultry and cheese among both sexes and in addition that of liquid dairy products among women was higher in higher climate impact tertiles. The consumption of red and processed meat in relation to energy in the lowest and highest climate impact tertiles was 8·2 g/MJ and 18·1 g/MJ in men, respectively, and 6·0 g/MJ and 13·5 g/MJ in women, respectively. By contrast, the consumption of vegetables and fruit as well as cereals was lower among higher dietary climate impact tertiles. The consumption of vegetables and fruit in relation to energy in the lowest and highest climate impact tertiles was 38 g/MJ and 30 g/MJ in men, respectively, and 58 g/MJ and 43 g/MJ in women, respectively.

### Macronutrient intake by dietary climate impact tertiles

The focus in the case of the intake of macronutrients in dietary climate impact tertiles was on the figures expressed as energy percentage (E%) and in relation to energy (g/MJ) for fibre. The intakes of energy (MJ/d) and protein (E%) were higher in higher dietary climate impact tertiles (Table 4). The intake of total fat (E%) did not differ by climate impact tertiles. The intake of SFA (E%) was higher in higher dietary climate impact tertiles with significant differences between all tertiles among women. Among men, the intake of saturated fat (E%) was higher in the middle tertile compared to the lowest tertile. The intake of carbohydrates (E%) and fibre (g/MJ) was lower in higher dietary climate impact tertiles. The intake of PUFA (E%) among women was lower in the highest dietary impact tertile compared to the lowest tertile. Among men, the intake of PUFA (E%) did not differ between the climate impact tertiles.

**Table 4:** Means and CIs of energy and macronutrient intakes in men and women by dietary climate impact (greenhouse gas emission sum) tertiles (FinDiet 2017 survey, *n* 1247, under-reporters excluded)

	Men									Women								
		Climate impact tertile									Climate impact tertile							
	All men	Lowest (1)		Middle (2)		Highest (3)		General test	Pairwise comparison	All women	Lowest (1)		Middle (2)		Highest (3)		General test	Pairwise comparison
	Mean	Mean	95 % CI	Mean	95 % CI	Mean	95 % CI	*P* value	Sign. diff.^*^	Mean	Mean	95 % CI	Mean	95 % CI	Mean	95 % CI	*P* value	Sign. diff.^*^
Energy (MJ)	10·5	8·7	8·4, 8·9	10·3	10·0, 10·6	12·2	11·7, 12·7	0·00001	1 < 2, 1 < 3, 2 < 3	8·1	7·0	6·9, 7·2	8·0	7·8, 8·2	9·1	8·9, 9·4	0·00001	1 < 2, 1 < 3, 2 < 3
Protein (E%)	17·7	16·0	15·5, 16·5	17·2	16·7, 17·6	19·5	18·8, 20·2	0·00001	1 < 2, 1 < 3, 2 < 3	17·0	15·9	15·4, 16·3	16·9	16·5, 17·4	17·9	17·3, 18·5	0·00001	1 < 2, 1 < 3, 2 < 3
Total carbohydrates (E%)	41·4	44·1	42·8, 45·4	41·4	40·5, 42·4	39·3	38·2, 40·4	0·0001	1 > 2, 1 > 3	42·3	44·0	43·1, 45·0	41·8	40·8, 42·8	41·1	40·3, 42·0	0·00001	1 > 2, 1 > 3
Fibre (g/MJ)	2·4	2·8	2·7, 3·0	2·4	2·3, 2·6	2·2	2·1, 2·3	0·00001	1 > 2, 1 > 3	2·8	3·2	3·0, 3·3	2·7	2·6, 2·9	2·5	2·4, 2·7	0·00001	1 > 2, 1 > 3
Fat (E%)	38·9	37·7	36·3, 39·0	39·5	38·5, 40·4	39·4	38·5, 40·4	NS		38·5	37·5	36·5, 38·5	39·1	38·0, 40·1	38·9	38·1, 39·8	NS	
SFA (E%)	15·2	14·6	14·0, 15·2	15·7	15·2, 16·3	15·3	14·7, 15·8	0·04	1 < 2	14·5	13·5	12·9, 14·0	14·9	14·4, 15·5	15·1	14·7, 15·5	0·00001	1 < 2, 1 < 3
PUFA (E%)	6·8	7·0	6·6, 7·3	6·8	6·5, 7·1	6·8	6·5, 7·1	NS		7·1	7·3	7·0, 7·7	7·1	6·8, 7·4	6·8	6·5, 7·1	0·02	1 > 3
*n*-3 PUFA (E%)	1·6	1·7	1·5, 1·8	1·5	1·4, 1·6	1·5	1·4, 1·6	0·04	NS	1·7	1·8	1·7, 2·0	1·7	1·6, 1·8	1·6	1·5, 1·7	NS	

Sign. diff., significant difference.

* Considered significantly different with group rankings as indicated, if for the general test *P* < 0·05 and for pair-wise comparison *P* < 0·05.

The intake of macronutrients as g/d is presented in online supplementary material, Supplemental Table 1. The intake (g/d) of protein, total carbohydrates, fat, SFA and PUFA was higher in higher climate impact tertiles. The intake of fibre (g/d) did not differ between the climate impact tertiles.

### Food ingredient groups as contributors to dietary climate impact

The contribution of different food ingredient groups to the total dietary climate impact by sex is presented in online supplementary material, Supplemental Table 2. Red and processed meat was a major contributor to dietary climate impact with a share of 40 % in men and 30 % in women. Within the red and processed meat group, beef was the main contributor to dietary climate impact. Beef contributed 20 % and 16 % of total climate impact in men and women, respectively.

The proportions of different food ingredient groups as contributors to total dietary climate impact varied between the climate impact tertiles (Figure 1). The share of red and processed meat was 49 % and 41 % in the highest tertile and 20 % and 15 % in the lowest tertile in men and women, respectively. On the other hand, the share of vegetables and fruit was 6 % and 9 % in the highest tertile and 10 % and 16 % in the lowest tertile in men and women, respectively. Some variation by climate impact tertiles was also seen for dairy products as contributors to total dietary climate impact with a share of 17 % and 19 % in the highest tertile and 26 % and 23 % in the lowest tertile in men and women, respectively. The share of poultry and fish and seafood as contributors to total dietary climate impact seemed quite even across the climate impact tertiles.

The results by age showed that red and processed meat contributed 43 % and 33 % in the youngest age group and 37 % and 28 % in the oldest age group of total dietary climate impact in men and women, respectively (see online supplementary material, Supplemental Figure 1).

## Discussion

Based on actual food consumption data from Finland, the dietary climate impact was lower among women than among men and lower among older age groups compared to younger age groups. The association between the consumption of different food ingredient groups and the dietary climate impact was somewhat different considering food consumption as absolute amounts (g/d) and in relation to energy (g/MJ). Our results support the conclusion of a recent study from Sweden that to comprehensively understand dietary sustainability, more than one analytical approach may be needed^(23)^. For several food groups, higher absolute consumption (g/d) was associated with higher climate impact. When food consumption was examined in relation to energy (g/MJ), the consumption of animal-based foods such as red and processed meat, poultry and cheese was associated with a higher dietary climate impact, whereas the consumption of plant-based foods such as vegetables and fruit as well as cereals was associated with a lower dietary climate impact. Presenting both approaches increases understanding of the different aspects of dietary climate impacts. The results based on absolute consumption provide a general picture of the associations, whereas results for food consumption in relation to energy illustrate the quality of diets in relation to their climate impact. In our study, results of macronutrient intakes in relation to energy (i.e. energy percentages or fibre as g/MJ) point to higher intakes of energy, protein and SFA (E%), and lower intakes of carbohydrates (E%) and fibre (g/MJ) in higher dietary climate impact tertiles. In general, food consumption and macronutrient intakes in the lowest dietary climate impact tertile were closer to the Nordic Nutrition recommendations^(6)^ than in the other tertiles.

The absolute dietary climate impact was higher in men than in women, which is in line with earlier literature^(13–16,21,23–26)^. The difference between the sexes is probably related both to the higher overall food consumption as well as differences in food choices, for example, almost twofold consumption of red and processed meat in men compared to women, according to our study. In our study, the dietary climate impact was lower among older age groups compared to younger age groups, which is in line with recent findings from Finland based on food frequency questionnaire data^(25)^, while inconsistency in results for age groups has generally been seen in previous studies^(28)^. Interestingly, our results also suggest that the share of red and processed meat as a contributor to dietary climate impact was highest in the youngest age group in both sexes. Thus, our results do not suggest that people in younger age groups would have adopted more plant-based diets and would try to avoid the consumption of red and processed meat. However, in our analyses, the youngest age group did not only include young adults, but the age range was quite wide: 18–44 years, as our data did not allow a finer age categorisation. In our study, no significant differences by education, income or urbanisation level were seen. However, in another Finnish study utilising food frequency questionnaire data and comprising a considerably larger sample than ours, sociodemographic differences in dietary climate impact were examined for several sociodemographic indicators^(25)^. The absolute dietary climate impact was higher among women living in remote rural areas compared to more urban living areas, whereas the dietary climate impact was the lowest in men in the lowest income quintile and the highest in men in the highest quintile. Differences by education were not observed.

Red and processed meat and dairy products were major contributors to the total dietary climate impact, supporting the findings from other studies^(21,25,26,29)^. In Finland, currently about 93 % of men and 60 % of women exceed the recommendation of a maximum of 350 g of red and processed meat per week, indicating a need for changes in diets^(9)^. We further examined the aspect of food ingredient groups as contributors to dietary climate impact by age and climate impact tertiles. In a study from the Netherlands, including participants aged seven and older, the contribution of meat and cheese to the dietary climate impact was 40 % in all included subgroups by sex and age, i.e. among girls, boys, women and men^(21)^. However, as our data only included adults, our results by age cannot be directly compared with the findings from the Netherlands. Overall, the results on food consumption and on the contributors of dietary climate impact by dietary climate impact tertiles highlight the marked role of red meat in high climate impact diets.

Under-reporting of energy intake is a well-founded concern in all dietary studies based on participants’ self-reports^(39)^. We examined under-reporting among all FinDiet 2017 Survey participants and about half of the subjects in the lowest dietary climate impact group were classified as energy under-reporters. To enable reasonable comparisons between dietary climate impact groups, we excluded energy under-reporters from the data. Exclusion of energy under-reporters may have resulted in misclassification of some subjects but was necessary because of the high proportion of under-reporters in the lowest dietary climate impact group. Exclusion of energy under-reporters also improved the reliability of the estimates of the actual dietary climate impact, as under-reporting of food consumption and thereby energy intake also leads to underestimates of the climate impact. Even after the exclusion of under-reporters, dietary climate impact increased along with energy intake, which is plausible. Higher share of under-reporters in lower dietary climate impact groups has also been seen in other studies^(21–23)^. Accounting for energy under-reporting in earlier studies seems inconsistent. Energy under-reporters have been excluded from the analyses in some studies in all analyses^(15,16)^ or in selected analyses^(24)^, whereas in other studies under-reporting has been accounted for by adjusting for estimated energy requirement^(29)^ or by comparing several approaches^(22)^.

The ways to account for the positive relationship between energy intake and dietary climate impact have varied as well. In some studies, food consumption and nutrient intakes have been adjusted for total energy intake^(13,15)^; in some studies, the dietary GHGEs have been adjusted for energy intake^(16,18)^, while in one study both of them were considered in relation to energy intake^(14)^. A study from Sweden, which focused on the relations between dietary GHGEs and micronutrient intake, compared several methods to adjust for energy intake: GHGE per day, GHGE per 1000 kcal as well as adjusting for energy intake by including total energy intake as a covariate in the statistical model or using the residual method^(23)^. We formed the dietary climate impact tertiles based on absolute dietary GHGE sums without adjustment for energy intake so that they would reflect real diets and the interpretation would remain as concrete as possible. Energy intake was accounted for in the food consumption and macronutrient intake calculations, which were explored both as absolute consumption (g/d) and in relation to energy (g/MJ or E%), reflecting the composition of diet. The contrasting results based on the two approaches confirm the relevance of thorough consideration of the association of energy intake and climate impact of diets in future studies and underline the need for an explicit and transparent description of how energy intake and under-reporting are accounted for. Considering the quality of diets is essential in the pursuit of decreasing dietary climate impacts, while on the other hand, in the real world, environmental impacts are related to actual diets.

The strengths of this study include the food consumption data from the National FinDiet 2017 Survey representing the Finnish adult population^(30)^ and the nationally produced climate impact coefficients obtained from the Natural Resources Institute Finland^(36)^. Another strength of our study is our approach. It included a thorough consideration of the role of energy intake, which broadens the understanding of the relationship between diets and their climate impacts.

Ideally, multiple sustainability indicators would be considered simultaneously in dietary analyses^(17)^. A limitation of our study was that, of the different dietary environmental impact indicators, we used only GHGEs, i.e. the climate impact. Climate impact is the most abundantly used indicator for dietary environmental impact in research literature^(3)^, but in future studies, it is relevant to shift the focus to the impacts on water use, eutrophication and biodiversity loss, among others. Our analyses also focused on food consumption and on macronutrients. In future studies, examining micronutrient intakes by dietary climate impact level would complement the current findings.

Our data are from year 2017, and possible changes in food consumption patterns thereafter in different population groups in Finland could not be taken into account. For example, the variety of plant-based foods has widened and the discussion on the environmental aspects of food consumption has increased in the Finnish society. More broadly, the COVID-19 pandemic and recent changes in the geopolitical situation in Europe have led to a greater focus on food security and may thereby also have affected food consumption.

Further, it is possible that all existing population group differences could not be observed because of the restrictions in the size of the data after the exclusion of energy under-reporters. It is essential to examine the population group differences when more recent dietary data from Finland is available with thorough consideration of energy intake also in the population group comparisons.

### Conclusions

Our study from Finland showed that the dietary climate impact was lower among women than among men and lower among older age groups compared to younger age groups. The relations between food consumption and macronutrient intake and the dietary climate impact were somewhat different when food consumption and macronutrient intake were considered as absolute amounts (g/d) compared to considering them in relation to energy (g/MJ or E%). In relation to energy intake, the consumption of animal-based foods was higher and that of plant-based foods lower in higher climate impact tertiles compared to lower tertiles. For macronutrient intakes, our results suggest more favourable diets with respect to the recommendations in lower dietary climate impact tertiles compared to higher tertiles. Higher intake of carbohydrates (E%) and fibre (g/MJ) as well as PUFA (E%) among women was associated with a lower dietary climate impact. Our results also suggest an association between higher SFA intake (E%) and higher dietary climate impact. Overall, our study emphasises consideration of the quality of diets, i.e. food consumption in relation to energy intake (g/MJ), not just absolute consumption (g/d), when the characteristics of diet in different environmental impact categories are studied. Our study showed that red and processed meat was a major contributor to dietary climate impact and that red and processed meat consumption was highest in the highest climate impact tertile.

Thus, at a research and policy level, it is essential to design and implement effective measures for dietary changes among the Finnish population towards the current Nordic and Finnish national nutrition recommendations^(6,9),^ as this would promote both healthier and more climate-friendly diets. As the dietary climate impact is higher among men than among women and also somewhat higher among adults aged 18–44 years than among older age groups, measures targeting these population groups are required especially. Examples of suitable measures might include, for instance, engaging young or early middle-aged parents at child health clinics or school health care with dietary advice directed to whole families or urging the food industry and the work catering services to develop or serve more plant-based foods that would be appealing also to working-age men.

## Supporting information

Paalanen et al. supplementary material Paalanen et al. supplementary material

## Supplementary material

For supplementary material accompanying this paper, visit https://doi.org/10.1017/S1368980025101730

## References

[1] Willett W , Rockström J , Loken B et al. (2019) Food in the Anthropocene: the EAT–Lancet Commission on healthy diets from sustainable food systems. Lancet 393, 447–492. 30660336 10.1016/S0140-6736(18)31788-4

[2] Bui LP , Pham TT , Wang F et al. (2024) Planetary Health Diet Index and risk of total and cause-specific mortality in three prospective cohorts. Am J Clin Nutr 120, 80–91. 38960579 10.1016/j.ajcnut.2024.03.019 PMC11251201

[3] Carey CN , Paquette M , Sahye-Pudaruth S et al. (2023) The environmental sustainability of plant-based dietary patterns: a scoping review. J Nutr 153, 857–869. 36809853 10.1016/j.tjnut.2023.02.001

[4] Trolle E , Meinilä J , Eneroth H et al. (2024) Integrating environmental sustainability into food-based dietary guidelines in the Nordic countries. Food Nutr Res 68, 1–15. doi: 10.29219/fnr.v68.10792 PMC11549731 39525324

[5] James-Martin G , Baird DL , Hendrie GA et al. (2022) Environmental sustainability in national food-based dietary guidelines: a global review. Lancet Planet Health 6, e977–986. 36495892 10.1016/S2542-5196(22)00246-7

[6] Blomhoff R , Andersen R , Arnesen EK et al. (2023) Nordic Nutrition Recommendations 2023. Copenhagen, Denmark: Nordic Council of Ministers.

[7] Schäfer AC , Boeing H , Gazan R et al. (2025) A methodological framework for deriving the German food-based dietary guidelines 2024: food groups, nutrient goals, and objective functions. Plos One 20, e0313347. 40073305 10.1371/journal.pone.0313347 PMC11903038

[8] World Health Organization, Food and Agriculture Organization of the United Nations (2019) Sustainable Healthy Diets – Guiding Principles. Rome, Italy: World Health Organization, Food and Agriculture Organization of the United Nations.

[9] National Nutrition Council and Finnish Institute for Health and Welfare (2024) Sustainable health from food - National Nutrition Recommendations 2024. Directions 15/2024. Helsinki, Finland: PunaMusta Oy.

[10] Tubiello FN , Rosenzweig C , Conchedda G et al. (2021) Greenhouse gas emissions from food systems: building the evidence base. Environ Res Lett 16, 065007.

[11] Crippa M , Solazzo E , Guizzardi D et al. (2021) Food systems are responsible for a third of global anthropogenic GHG emissions. Nat Food 2, 198–209. 37117443 10.1038/s43016-021-00225-9

[12] Tubiello FN , Karl K , Flammini A et al. (2022) Pre- and post-production processes increasingly dominate greenhouse gas emissions from agri-food systems. Earth Syst Sci Data 14, 1795–1809.

[13] Monsivais P , Scarborough P , Lloyd T et al. (2015) Greater accordance with the Dietary Approaches to Stop Hypertension dietary pattern is associated with lower diet-related greenhouse gas production but higher dietary costs in the United Kingdom. Am J Clin Nutr 102, 138–145. 25926505 10.3945/ajcn.114.090639 PMC4480663

[14] Rose D , Heller MC , Willits-Smith AM et al. (2019) Carbon footprint of self-selected US diets: nutritional, demographic, and behavioral correlates. Am J Clin Nutr 109, 526–534. 30698631 10.1093/ajcn/nqy327 PMC6408204

[15] Seconda L , Baudry J , Allès B et al. (2018) Comparing nutritional, economic, and environmental performances of diets according to their levels of greenhouse gas emissions. Clim Change 148, 155–172.

[16] Sjörs C , Hedenus F , Sjölander A et al. (2017) Adherence to dietary recommendations for Swedish adults across categories of greenhouse gas emissions from food. Public Health Nutr 20, 3381–3393. 28879831 10.1017/S1368980017002300 PMC10261478

[17] Mazac R , Hyyrynen M , Kaartinen NE et al. (2024) Exploring tradeoffs among diet quality and environmental impacts in self-selected diets: a population-based study. Eur J Nutr 63, 1663–1678. 38584247 10.1007/s00394-024-03366-2 PMC11329690

[18] Sugimoto M , Murakami K , Fujiwara A et al. (2020) Association between diet-related greenhouse gas emissions and nutrient intake adequacy among Japanese adults. Plos One 15, e0240803. 33095787 10.1371/journal.pone.0240803 PMC7584234

[19] Vieux F , Soler LG , Touazi D et al. (2013) High nutritional quality is not associated with low greenhouse gas emissions in self-selected diets of French adults. Am J Clin Nutr 97, 569–583. 23364012 10.3945/ajcn.112.035105

[20] Saarinen M , Pellinen T , Kostensalo J et al. (2025) Dietary climate impact correlates ambiguously with health biomarkers– a randomised controlled trial in healthy Finnish adults. Eur J Nutr 64, 95. 39964546 10.1007/s00394-025-03609-w PMC11836174

[21] Temme EH , Toxopeus IB , Kramer GF et al. (2015) Greenhouse gas emission of diets in the Netherlands and associations with food, energy and macronutrient intakes. Public Health Nutr 18, 2433–2445. 25543460 10.1017/S1368980014002821 PMC10271514

[22] Murakami K & Livingstone MBE (2018) Greenhouse gas emissions of self-selected diets in the UK and their association with diet quality: is energy under-reporting a problem? Nutr J 17, 27. 29466993 10.1186/s12937-018-0338-x PMC5822528

[23] Stubbendorff A , Hallström E , Tomova G et al. (2025) Greenhouse gas emissions in relation to micronutrient intake and implications of energy intake: a comparative analysis of different modeling approaches. Am J Clin Nutr 121, 1063–1076. 40074038 10.1016/j.ajcnut.2025.02.031 PMC12107493

[24] Hyland JJ , Henchion M , McCarthy M et al. (2017) The climatic impact of food consumption in a representative sample of Irish adults and implications for food and nutrition policy. Public Health Nutr 20, 726–738. 27667716 10.1017/S1368980016002573 PMC10261633

[25] Sares-Jäske L , Härkänen T , Tapanainen H et al. (2025) Sociodemographic and regional differences in dietary climate impact: findings from Finnish population surveys. Front Sustain Food Syst 9. doi: 10.3389/fsufs.2025.1543646.

[26] Lengle JM , Michaelsen Bjøntegaard M , Carlsen MH et al. (2024) Environmental impact of Norwegian self-selected diets: comparing current intake with national dietary guidelines and EAT-Lancet targets. Public Health Nutr 27, e100. 38523532 10.1017/S1368980024000715 PMC11010176

[27] van Bussel LM , van Rossum CT , Temme EH et al. (2020) Educational differences in healthy, environmentally sustainable and safe food consumption among adults in the Netherlands. Public Health Nutr 23, 2057–2067. 32383426 10.1017/S1368980019005214 PMC10200583

[28] Kliejunas E , Cleghorn C , Drew JM et al. (2025) The relationship between dietary greenhouse gas emissions and demographic characteristics in high-income countries. Proc Nutr Soc 84, 139–147. 39558471 10.1017/S0029665124007523

[29] Guðmannsdóttir R , Gunnarsdóttir S , Geirsdóttir ÓG et al. (2024) Greenhouse gas emissions of environmentally sustainable diets: insights from the Icelandic National Dietary Survey 2019–2021. J Clean Prod 467, 142906.

[30] Kaartinen N , Tapanainen H , Reinivuo H et al. (2020) The Finnish national dietary survey in adults and elderly (FinDiet 2017). EFSA Support Publ 17, 1914E.

[31] Koponen P , Borodulin K , Lundqvist A et al. (2018) *Health, Functional Capacity and Welfare in Finland – FinHealth 2017 Study (in Finnish). Report 4/2018*. Helsinki, Finland: Finnish Institute for Health and Welfare.

[32] Borodulin K & Sääksjärvi K (editors) (2019) *FinHealth 2017 Study – Methods. Report 17/2019*. Helsinki, Finland: Finnish Institute for Health and Welfare.

[33] Finnish Institute for Health and Welfare (2018) National Food Composition Database FINELI®, Release 19. https://fineli.fi/fineli/en/index? (accessed December 2024).

[34] Reinivuo H , Hirvonen T , Ovaskainen ML et al. (2010) Dietary survey methodology of FINDIET 2007 with a risk assessment perspective. Public Health Nutr 13, 915–919. 20513260 10.1017/S1368980010001096

[35] European Food Safety Authority (2014) Guidance on the EU Menu methodology. EFSA J 12, 3944.

[36] Saarinen M , Heikkinen J , Ketoja E et al. (2023) Soil carbon plays a role in the climate impact of diet and its mitigation: the Finnish case. Front Sustain Food Syst 7. doi: 10.3389/fsufs.2023.904570

[37] OECD Project on Income Distribution and Poverty (2021) Glossary: Equivalised Disposable Income. https://ec.europa.eu/eurostat/statistics-explained/index.php?title=Glossary:Equivalised_disposable_income (accessed June 2025).

[38] Härkänen T , Karvanen J , Tolonen H et al. (2016) Systematic handling of missing data in complex study designs – experiences from the Health 2000 and 2011 Surveys. J Appl Stat 43, 2772–2790.

[39] Macdiarmid J & Blundell J (1998) Assessing dietary intake: who, what and why of under-reporting. Nutr Res Rev 11, 231–253. 19094249 10.1079/NRR19980017

