# Supplementary material for: The association of food consumption and macronutrient intake with dietary climate impact in Finland: considerations on the role of energy intake
Source: Public Health Nutr. 2026 Jan 2;29(1):e21. doi: 10.1017/S1368980025101730 (PMC12895455; doi:10.1017/S1368980025101730)
Supplement: Paalanen et al. supplementary material [file S1368980025101730sup001.pdf]

The association of food consumption and macronutrient intake with dietary climate impact in Finland: considerations on the role of energy intake

Supplementary material

Supplementary Table 1. Means and confidence intervals (CI) of macronutrient intakes (g/day) in men and women by dietary climate impact (greenhouse gas emission sum) tertiles (FinDiet 2017 Survey, n=1247, under-reporters excluded).

	**Men (g/day)**									**Women (g/day)**								
		Climate impact tertile									Climate impact tertile							
	All men	Lowest (1)		Middle (2)		Highest (3)		General test	Pairwise comparison	All women	Lowest (1)		Middle (2)		Highest (3)		General test	Pairwise comparison
	Mean	Mean	95% CI	Mean	95% CI	Mean	95% CI	p value	Sign. diff.*	Mean	Mean	95% CI	Mean	95% CI	Mean	95% CI	p value	Sign. diff.*
Protein	107.7	79.8	76.3, 83.3	101.0	97.5, 104.5	134.7	127.2, 142.2	<0.001	2>1, 3>1, 3>2	79.0	64.5	62.7, 66.4	77.6	75.5, 79.7	92.9	89.9, 95.9	<0.001	2>1, 3>1, 3>2
Total carbohydrates	245	216	208, 224	241	230, 252	270	254, 286	<0.001	2>1, 3>1, 3>2	194	177	171, 182	190	184, 197	212	204, 220	<0.001	3>1, 3>2
Fibre	24.6	24.0	22.8, 25.3	24.3	22.4, 26.1	25.4	24.0, 26.8	NS		22.3	22.5	21.0, 23.9	21.7	20.4, 23.0	22.6	21.3, 23.9	NS	
Fat	108.7	86.7	82.5, 90.9	108.0	103.1, 112.9	126.1	119.8, 132.4	<0.001	2>1, 3>1, 3>2	83.7	70.8	68.3, 73.4	83.7	80.8, 86.7	94.9	91.1, 98.7	<0.001	2>1, 3>1, 3>2
Saturated fatty acids	42.4	33.5	31.8, 35.2	43.1	40.9, 45.2	48.7	46.6, 50.8	<0.001	2>1, 3>1, 3>2	31.6	25.3	24.2, 26.3	32.0	30.6, 33.3	36.8	35.1, 38.5	<0.001	2>1, 3>1, 3>2
Polyunsaturated fatty acids	19.1	16.0	15.0, 17.0	18.7	17.6, 19.8	21.9	20.1, 23.6	<0.001	2>1, 3>1, 3>2	15.3	14.0	13.0, 15.1	15.1	14.4, 15.9	16.6	15.7, 17.5	0.002	3>1
N-3 polyunsaturated fatty acids	4.3	3.9	3.5, 4.2	4.1	3.9, 4.4	4.8	4.3, 5.3	0.009	3>1	3.7	3.5	3.1, 3.9	3.6	3.4, 3.9	3.9	3.6, 4.2	NS	

CI, confidence interval; Sign. diff., significant difference; NS, non-significant

*Considered significantly different with group rankings as indicated, if for the general test p<0.05 and for pair-wise comparison p<0.05.

Supplementary Table 2. The proportion of food ingredient groups as contributors of dietary climate impact by sex (FinDiet 2017 Survey, n=1247, under-reporters excluded).

	Men (%)	Women (%)
Vegetables and fruit*	7.1	11.8
Potatoes	0.3	0.2
Legumes	0.5	0.7
Nuts and seeds	0.2	0.3
Red and processed meat	39.7	30.1
Beef	20.1	15.8
Pork	3.7	2.5
Other red meat or processed meat	15.9	11.8
Poultry	4.7	5.0
Fish and seafood	2.9	3.1
Liquid dairy products	7.9	8.9
Cheese†	8.8	8.1
Butter and butter-containing spreads	3.9	3.8
Vegetable oil and margarine	2.1	2.0
Cereals‡	4.1	4.2
Other	17.9	21.6

*Including berries.

†Only matured cheeses included.

‡Including wheat, barley, oat, rye, rice, starch, other cereals and cereal bars.

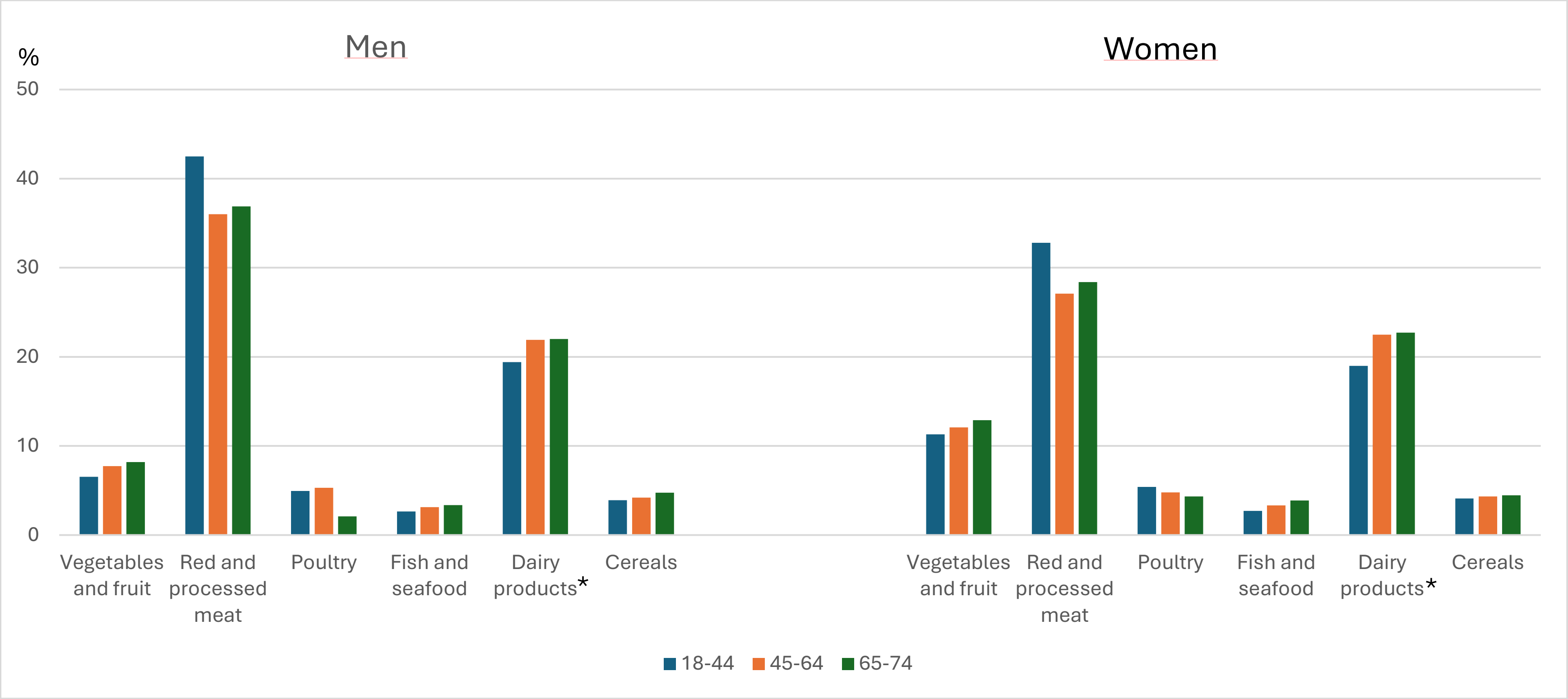

*Dairy products are a sum of liquid dairy products, cheese (matured cheeses) and butter and butter-containing spreads.

Supplementary Figure 1. Proportion of food ingredient groups as contributors of dietary climate impact in three age groups by sex (FinDiet 2017 Study, n=1247, under-reporters excluded).
